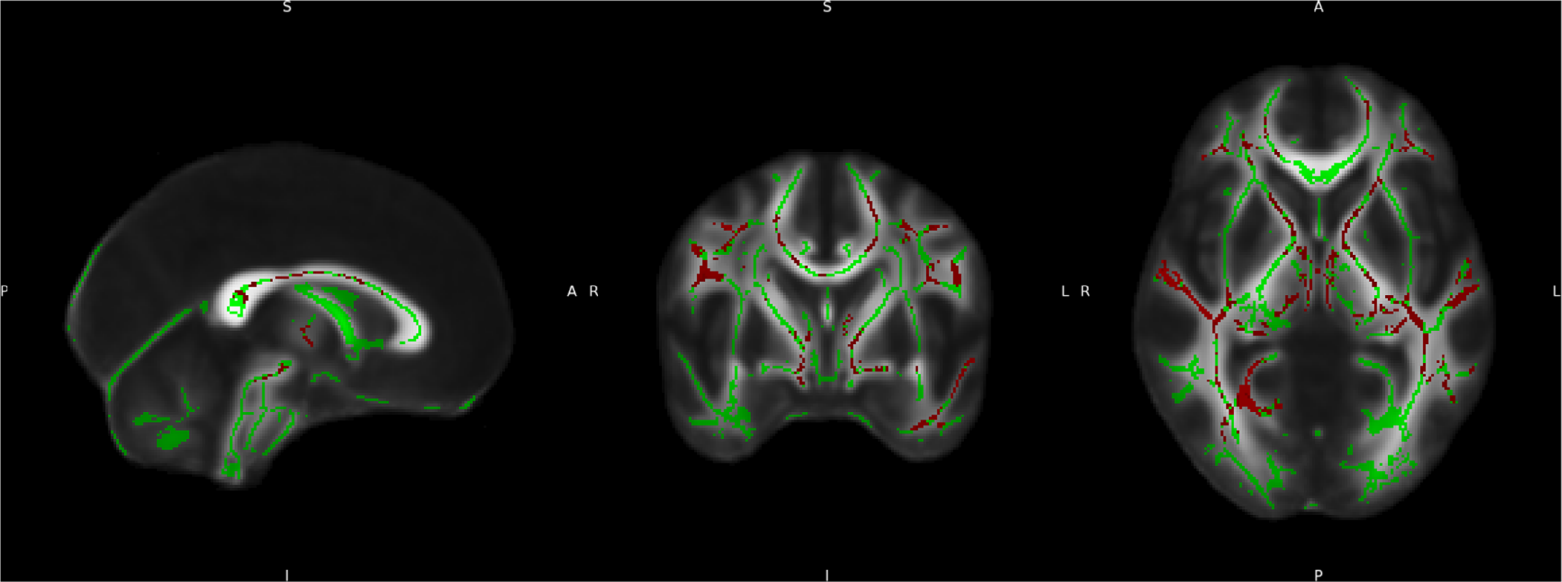# White matter integrity predicts executive dysfunction in later‐life attention deficit/hyperactivity disorder

**DOI:** 10.1002/alz70857_104000

**Published:** 2025-12-24

**Authors:** Natalia Docteur, S. Benjamin Peckham, Hayley Huston, Sara Becker, Lanea Jackson, Andrew E. Beaudin, Ryan T. Muir, Eric E. Smith, Jodie Gawryluk, Brandy L. Callahan

**Affiliations:** ^1^ University of Calgary, Calgary, AB, Canada; ^2^ Hotchkiss Brain Institute, Calgary, AB, Canada; ^3^ University of Victoria, Victoria, BC, Canada

## Abstract

**Background:**

Attention deficit/hyperactivity disorder (ADHD) is a risk factor for cognitive decline and dementia, in particular vascular and Lewy Body subtypes. The reasons for this are unclear. White matter integrity (WMI) in ADHD is characterized by delayed and deficient tract development compared to healthy controls and predicts reduced cognitive performance. Impaired WMI in ADHD may be a marker of neurobiological vulnerability to subsequent injury from both healthy and degenerative brain aging processes, resulting in increased dementia risk. We investigated the relationship between WMI and cognition in adults with ADHD while accounting for age.

**Method:**

Adults aged >40 with ADHD were recruited from the community for this cross‐sectional observational study. ADHD diagnosis was confirmed through structured clinical interview. All participants completed life history interviews, clinical questionnaires, cognitive testing, and structural neuroimaging that included diffusion‐weighted imaging sequences. Whole‐brain fractional anisotropy (FA) was calculated as a metric of WMI using tract‐based spatial statistics. Cognitive outcomes were mean *z*‐score composites for executive function, processing speed, immediate recall, and delayed recall. The relationships between FA and cognitive performance, covarying for age, were examined using general linear models corrected for multiple comparisons.

**Result:**

Analyses included 45 participants with ADHD aged 58.5 ± 10.3 years (range: 40‐80), 64.4% female, education =  15.2 ± 2.5 years, and 82.2% White. FA was significantly and positively associated with executive function while accounting for age in several white matter tracts throughout the brain. These included the cingulum, anterior thalamic radiations, uncinate fasciculi, corpus callosum, forceps minor, anterior and superior corona radiata, inferior and superior longitudinal fasciculi, and inferior fronto‐occipital fasciculi (Figure 1). There were no significant relationships between FA and composites of processing speed, immediate recall, or delayed recall.

**Conclusion:**

Impaired WMI in diffuse tracts that support executive function in ADHD may contribute to increased risk for cognitive decline and dementias that present with primary executive dysfunction. Future research should examine neurobiological and lifestyle factors that may interact with decreased WMI in ADHD to augment dementia vulnerability. Identification of deficient WMI as a unique contributor to poor cognitive aging in ADHD may lead to targeted preventative interventions for this neurodiverse, high‐risk group.